# Stability, biomechanics and biocompatibility analysis following different preparation strategies of hierarchical zeolite coatings on titanium alloy surfaces

**DOI:** 10.3389/fbioe.2023.1337709

**Published:** 2023-12-22

**Authors:** Chang Liu, Jiaxin Zhang, Xin Zhao, Mingwei Xu, He Liu, Hongming Zhou

**Affiliations:** ^1^ School of Materials Science and Engineering, Central South University, Changsha, China; ^2^ Department of Orthopedics, The Second Hospital of Jilin University, Changchun, China

**Keywords:** biomechanics, biocompatibility, coating stability, hierarchical mesoporous zeolite, surface modification, titanium alloy

## Abstract

Traditional titanium alloy implant surfaces are inherently smooth and often lack effective osteoinductive properties. To overcome these limitations, coating technologies are frequently employed to enhance the efficiency of bone integration at the implant–host bone interface. Hierarchical zeolites, characterized by their chemical stability, can be applied to 3D-printed porous titanium alloy (pTi) surfaces as coating. The resulting novel implants with a “microporous-mesoporous-macroporous” spatial gradient structure can influence the behavior of adjacent cells; thereby, promoting the integration of bone at the implant interface. Consequently, a thorough exploration of various preparation methods is warranted for hierarchical zeolite coatings with respect to biocompatibility, coating stability, and osteogenesis. In this study, we employed three methods: *in situ* crystal growth, secondary growth, and layer-by-layer assembly, to construct hierarchical zeolite coatings on pTi, resulting in the development of a gradient structure. The findings of this investigation unequivocally demonstrated that the LBL-coating method consistently produced coatings characterized by superior uniformity, heightened surface roughness, and increased hydrophilicity, as well as increased biomechanical properties. These advantages considerably amplified cell adhesion, spreading, osteogenic differentiation, and mineralization of MC3T3-E1 cells, presenting superior biological functionality when compared to alternative coating methods. The outcomes of this research provide a solid theoretical basis for the clinical translation of hierarchical zeolite coatings in surface modifications for orthopedic implants.

## 1 Introduction

Titanium and its alloys, such as Ti6Al4V, are preferred metallic materials for orthopedic implants due to their high mechanical strength, excellent corrosion resistance, and good biocompatibility, offering broad prospects for medical applications (Mei et al., 2014; Zhang et al., 2017). However, their relatively high elastic modulus can lead to postoperative complications such as stress shielding-induced bone resorption (Ma et al., 2023). Therefore, 3D-printed porous titanium alloy implants (pTi) have been developed to address these issues by reducing the elastic modulus while maintaining mechanical strength (Yook et al., 2012; Wang et al., 2019). Additionally, pTi provides a “macroporous” structure, characterized by its controllable pore size, porosity, and interconnectivity (Wang et al., 2019). These features mimic the natural bone structure, thereby facilitating cell migration, vascularization, and bone formation. Nonetheless, pTi still exhibits a certain degree of biological inertness and poor osseoinductivity (Contaldo et al., 2021). The osseointegration between titanium alloys and host bone tissue remains a concern, and long-term clinical use has shown a lack of direct contact between bone and titanium implants (Geetha et al., 2009).

Enhancing the osseointegration of implants can be achieved through surface modification, involving the creation of coatings with precise structural, and physicochemical attributes (Ehlert et al., 2019). The successful realization of the biological efficacy of implants primarily hinges upon the biocompatibility and stability of coatings and the influence of coating microstructure on the functionality of surrounding cells (Bose and Tarafder, 2012; Scarano et al., 2018; Alshawwa et al., 2022). Zeolites are solid crystalline aluminosilicates characterized by their uniform microporous structures (Wang et al., 2020). These microporous zeolites are composed of a three-dimensional framework consisting of TO_4_ tetrahedra, where T represents either Si or Al. These tetrahedra are interconnected through shared oxygen ions, giving rise to a framework with pores and cavities of molecular dimensions that are evenly distributed (Gao and Zhang, 2020; Aljama et al., 2022). It is worth noting that each [AlO_4_]^5−^ tetrahedron carries a negative charge, which is balanced by cations that can be readily exchanged with other cations, such as Ag, Sr, and Ca, through ion-exchange processes (Amiripour and Ghasemi, 2018; Yong et al., 2022).

In recent years, microporous zeolites and their coatings have attracted widespread attention and application across various domains of biomedicine and bone tissue engineering due to their excellent physicochemical properties, such as biocompatibility, ion-exchange capacity, and biological stability (Pan et al., 2017; Wang et al., 2019; Khojaewa et al., 2019). Wang et al. (Wang et al., 2018) employed an *in situ* growth method to fabricate a microporous zeolite coating on the surface of porous titanium implants and confirmed that the zeolite coating exhibited superior biocompatibility compared to bare implants. The zeolite coating was also found to stimulate the expression of osteogenic genes and promote new bone formation. In addition, the stability of surface coatings on implants plays a crucial role in the long-term realization of their biological functionality and the harnessing of their highly corrosion-resistant capabilities (Bacakova et al., 2018). Given that the elastic modulus of the zeolite coating falls within the range of 30–40 GPa, which is closely aligned with the elastic modulus of the host bone (approximately 30 GPa), a stable zeolite coating can effectively mitigate the resorption of implant materials. Furthermore, a stable zeolite coating can impede the release of toxic Al and V ions from the substrate titanium alloy, even after exposure to highly corrosive solutions (Bedi et al., 2009; Wang et al., 2018). At the same time, it is well established that surface topography significantly influences the regulation of cellular behaviors and functions. Qiao et al. (Qiao et al., 2020) highlighted the pivotal role of coating microtopography in modulating cellular bioactivity and differentiation capacity within the vicinity of the implant. Compared to bare implants, zeolite coatings enhance the adhesion, spreading, and proliferation capabilities of surrounding cells, such as bone marrow mesenchymal stem cells, osteoprogenitor cells, and osteoblasts (Li et al., 2015; Bacakova et al., 2018). This is primarily attributed to the 3D structure of the zeolite coatings.

In recent years, researchers have developed hierarchical zeolites with gradient variations in their microstructural morphology by subjecting zeolites to processes such as desilication and recrystallization (Gackowski and Datka, 2020). Compared to traditional microporous zeolites, hierarchical zeolites retain the framework structure of microporous zeolites while introducing larger mesoporous structures (2–50 nm) and macropores (greater than 50 nm) (Lin et al., 2018; Ghoneim et al., 2019). Furthermore, current trends in the field of implant morphology modification emphasize that hierarchical structures can further enhance cell adhesion and spreading (Gittens et al., 2014; Haïat et al., 2014). The design of gradient structure surface morphology, resembling natural bone tissue, demonstrates significant potential to enhance cellular functions from a biomimetic perspective (Ding et al., 2018). Thus, we contend that the construction of hierarchical zeolite coatings, in addition to the aforementioned advantages, also establishes a coating with a spatially graded structure of “microporous–mesoporous” on the implant surface. This will improve the biocompatibility and biological functionality of the coating, further promoting osseointegration and bone regeneration at the implant–host bone interface. However, current research mostly focuses on microporous zeolite coatings, and there is still a lack of research on hierarchical zeolite coatings.

**SCHEME 1:**
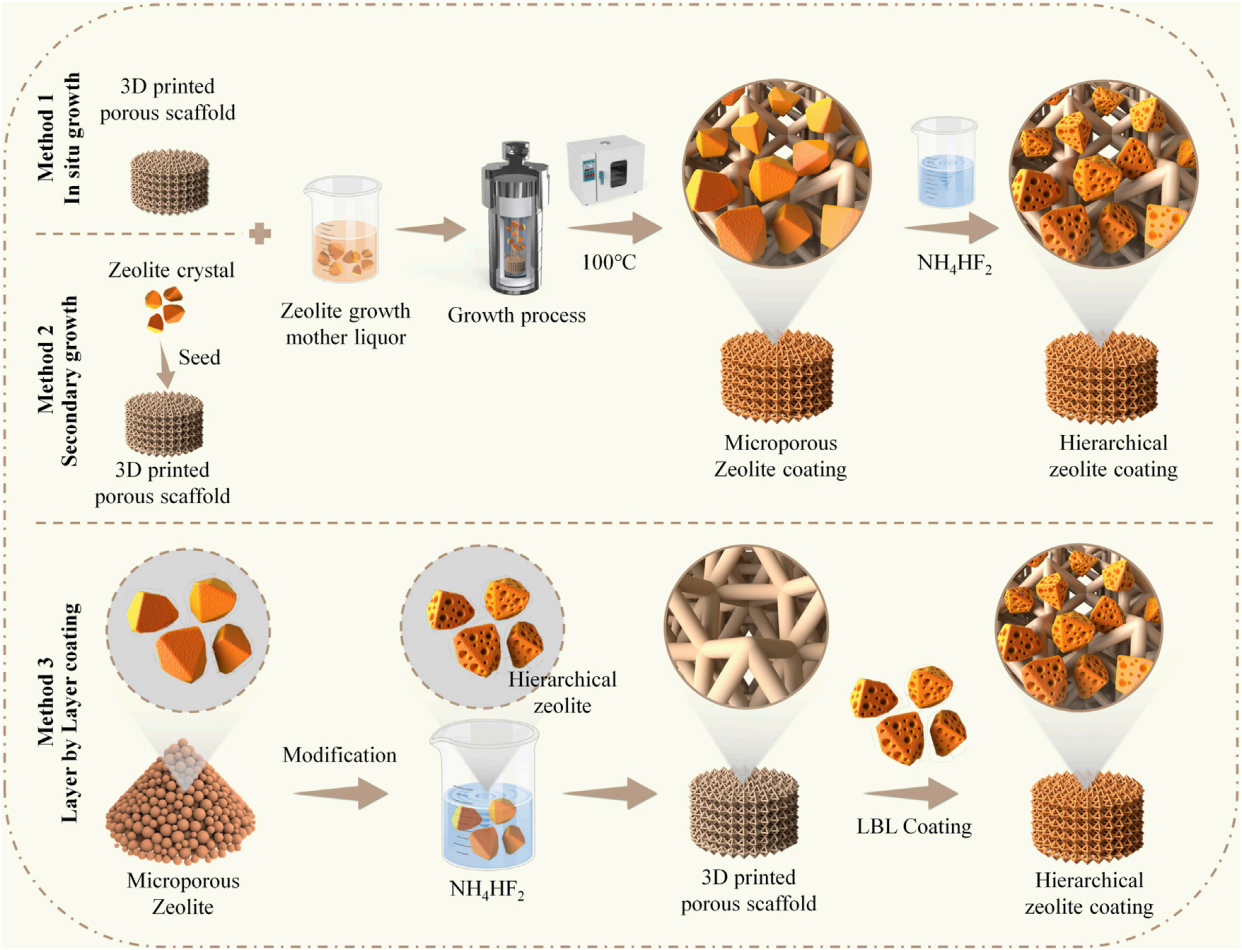
Schematic illustration demonstrates the construction of hierarchical zeolite coatings on the surface of 3D-printed titanium alloy porous implants utilizing three different methods.

In this work, we fabricated zeolite-A coatings on pTi using *in situ* growth and secondary growth methods, followed by a dealumination reaction with NH_4_HF_2_ solution. Furthermore, we employed an alternative layer-by-layer (LBL) electrostatic assembly method to construct hierarchical zeolite coatings (Scheme 1). Subsequently, we analyzed the physicochemical properties and stability of the coating products from the three methods used for coating construction. Simultaneously, we evaluated the biocompatibility of different coatings and assessed the impact of various surface microstructures on the adhesion, spreading, and osteogenic potential of MC3T3-E1 preosteoblasts. To the best of our knowledge, this is the first study to construct hierarchical zeolite coatings on 3D-printed titanium alloy implants using three different methods, establishing a structure-performance-effect relationship for implant coatings, which holds significant clinical implications.

## 2 Materials and methods

### 2.1 Materials and chemical reagents

3D-Printed porous Ti6Al4V scaffolds (diameter: 10 mm, height: 3 mm) were produced using the electron beam melting machine (EBM Q10 Plus, Arcam AB, United States). 4A zeolites were purchased from AOS Catalytic Materials Co., Ltd. (China); NaSiO_3_•9H_2_O from Xilong Scientific (China); NaAlO_2_ from Macklin (China); ammonium bifluoride (NH_4_HF_2_) from Aladdin Reagent Co., Ltd. (China); poly dimethyl diallyl ammonium chloride solution (PDDA) from Aladdin (China); and polyacrylic acid (PAA) from Acros Organic (Belgium). Dulbecco’s Modified Eagle’s Medium (DMEM)/F-12 and fetal bovine serum (FBS) were purchased from Gibco (United States). Phosphate-buffered saline (PBS), 4% paraformaldehyde, and phalloidin-FITC were purchased from Bioss (China). Calcein acetoxymethyl ester (Calcein-AM)/propidium iodide (PI) staining kit was purchased from Bestbio (China) and 4′,6-diamidino-2-phenylindole (DAPI) from Sigma–Aldrich (St. Louis, MO).

### 2.2 Preparation of the hierarchical zeolite coating

Before use, the pTi substrates underwent treatment in 30% H_2_O_2_ for a duration of 30 min. They were subsequently rinsed three times with deionized water and dried. Then, we utilized three methods to construct the hierarchical zeolite coatings.

#### 2.2.1 *In situ* hydrothermal growth and modification

The microporous zeolite coatings on the pTi surface were fabricated utilizing a *in situ* hydrothermal crystallization method. A clear silica source solution was prepared by mixing sodium silicate nonahydrate (NaSiO_3_•9H_2_O) and water in the specified proportion. Similarly, a clear aluminum source solution (NaAlO_2_) was prepared by mixing sodium aluminate and water in the specified ratio. The aluminum source solution was gradually added to the silica source solution with continuous stirring for 6 h, resulting in the formation of the mother liquor for the growth of microporous zeolite coatings. The molar ratio of components and solvents in the microporous zeolite coating growth mother liquor was as follows: 3.2Na_2_O:2SiO_2_:Al_2_O_3_:128H_2_O. The zeolite coatings were deposited on pretreated pTi after hydrothermal growth at 100°C for 12 h; then, the pTi was washed with distilled water in an ultrasonic bath and dried at 60°C for 6 h. Next, the pTi-microporous zeolite coating underwent a dealumination reaction in a 0.2 M NH_4_HF_2_ solution at 45°C for 2 h, leading to the creation of the hierarchical zeolite coating. It was subsequently rinsed with distilled water and dried at 60°C for 4 h and is denoted as **pTi-*in situ* growth**.

#### 2.2.2 Secondary growth and modification

The microporous zeolite coatings were synthesized on the pTi surface using a secondary growth method. Briefly, the pretreated pTi samples were alternately immersed in solutions of PDDA and 4A zeolite seed crystal growth mother liquor (both at 1 mg/mL) for 10 min, rinsed with deionized water, and then dried. Repeating the process five times yielded pTi substrates loaded with zeolite seed crystals. Subsequently, the growth mother liquor from the previous Section 2.2.1, which had a molar ratio of components and solvents of 3.2Na_2_O:2SiO_2_:Al_2_O_3_:128H_2_O, was employed for the preparation of the microporous zeolite coating. The zeolite coatings were deposited onto pretreated pTi at 100°C over 12 h. Following this, the pTi substrates were subjected to ultrasonic washing with distilled water and subsequently dried at 60°C for 6 h. Next, the microporous zeolite coating underwent a dealumination reaction in a 0.2 M NH_4_HF_2_ solution at 45°C for 2 h. It was subsequently rinsed with distilled water and dried at 60°C for 4 h and is denoted as **pTi-secondary growth**.

#### 2.2.3 LBL electrostatic assembly method

The hierarchical zeolites were generated by etching 4A zeolites (5 g) with NH_4_HF_2_ (0.2 M). The mixture was then washed using deionized water and centrifuged until the suspension reached a nearly neutral pH. The sample was then vacuum-dried at 80°C for 12 h. Subsequently, the pretreated pTi samples underwent sequential immersion in PDDA and PAA solutions (both at a concentration of 1 mg/mL) for 10 min, followed by rinsing with deionized water and drying. This process was repeated four times. The samples were then immersed alternately in PDDA and hierarchical zeolite solutions (both at a concentration of 1 mg/mL), followed by rinsing with deionized water and drying. This PDDA/Zeolite cycle was repeated four times. Finally, to eliminate organic compounds and enhance coating adhesion, the samples were calcined in a muffle furnace at 500°C for 4 h and are denoted as **pTi-LBL coating**.

#### 2.2.4 Surface characterization of different coatings

The integrity, homogeneity, particle distribution, and surface chemical composition of the coatings were assessed using scanning electron microscopy (SEM, ZEISS GeminiSEM 300, Germany). The surface microstructure and roughness were examined via atomic force microscopy (AFM, Bruker Dimension Icon, Germany). Water contact angles were measured with a contact angle-measuring instrument (Dataphysics OCA200, Germany). Pore size distribution and porosity were determined from N_2_ adsorption-desorption isotherms using a Micromeritics ASAP 2460 adsorption analyzer (United States).

### 2.3 Stability performance testing of the coatings and implants

To assess the stability of hierarchical zeolite coatings prepared using different construction methods, an innovative approach was adopted by integrating an ultrasonic oscillator (Bilon, Shanghai) with SEM. In brief, the pTi surface coatings constructed by three different methods were placed in the ultrasonic oscillator, operating continuously at an intensity of 50 kHz for 5 min. Subsequently, the sonicated pTi samples were dried at 60°C for 4 h. By utilizing SEM, the morphology and area of surface coatings were observed for the three sets of non-sonicated hierarchical zeolite coatings and the three sets of sonicated hierarchical zeolite coatings on pTi. This comparative analysis enabled an assessment of coating detachment, thereby determining the impact of the three different methods on coating stability. Images were analyzed using ImageJ (NIH, Bethesda, MD, United States). The compressive strength was tested using an electronic universal testing machine (WDW-100E) with a 10 kN load and a displacement speed of 5 mm/min. The relationship between stress and strain was determined by gradually increasing the pressure in the direction of the vertical axis. The maximum stress value in the curve was the ultimate compressive strength.

### 2.4 Biocompatibility evaluation of coatings

MC3T3-E1 preosteoblasts were purchased from Pu-nuo-sai Life Technology Co. Ltd. (Wuhan, China). MC3T3-E1 cells were cultured in DMEM/F12 containing 10% FBS, 1% penicillin/streptomycin, and incubated at 37°C under 5% CO_2_. Cells were seeded on pTi, pTi-*in situ* growth, pTi-secondary growth, and pTi-LBL coatings in 24-well plates at 2 × 10^4^ cells/well. After culturing for 1 day, a calcein-AM/PI working solution was prepared according to the manufacturer’s guidelines. The samples were then incubated in the working solution for 15 min at 37°C in the dark and then washed twice with PBS before being imaged under a fluorescence microscope (ECHO Revolve, United States). The images were analyzed using ImageJ.

### 2.5 Cell attachment and morphology on coated pTi surfaces

MC3T3-E1 preosteoblasts were cultured for 1 day in the presence of pTi, pTi-*in situ* growth, pTi-secondary growth, and pTi-LBL coatings in 24-well plates at a density of 2 × 10^4^ cells/well. After incubating for 1 day, the cells were then rinsed with PBS and fixed with 4% paraformaldehyde for 30 min at room temperature. The cells were then permeabilized with 0.5% Triton X-100 for 10 min followed by cytoskeletal and nuclear staining with phalloidin (30 min) and DAPI (30 s), respectively. The stained cells were then examined under a fluorescence microscope. The pTi, pTi-*in situ* growth, pTi-secondary growth, and pTi-LBL coating samples were placed in a 24-well cell culture plate. MC3T3-E1 cells were seeded onto the samples at a concentration of 2 × 10^4^/mL. After 12 and 24 h of incubation, gentle rinsing with PBS was performed, followed by fixation of cells and samples for 20 min. Subsequently, cell nuclei were stained with DAPI and observed under a fluorescence microscope, followed by image capture. The analysis aimed to assess the impact of the samples on cell adhesion. The images were analyzed using ImageJ.

The cells seeded on the pTi, pTi-*in situ* growth, pTi-secondary growth, pTi-LBL coatings were then washed with PBS and fixed by incubating in 2.5% (v/v) glutaraldehyde for 3 days. The samples were then dehydrated using a gradient series of ethanol solutions (30%, 50%, 70%, 80%, 90%, 95%, and 100%). They were then treated with a mixture of ethanol and isobutyl acetate at a 1:1 (v/v) ratio, followed by incubation in pure isobutyl acetate overnight. After critical point drying, the morphology of the cells adherent on the samples was examined using SEM.

### 2.6 Alkaline phosphatase and Alizarin Red S staining

MC3T3-E1 cells were co-cultured with pTi, pTi-*in situ* growth, pTi-secondary growth, and pTi-LBL coatings in 24-well plates in an osteogenesis differentiation medium (DMEM/F-12 containing 10% FBS, 50 μM vitamin C, 10 mM β-glycerol-phosphate, 0.1 μM dexamethasone, and 1% penicillin-streptomycin). After culture for 7 days, the alkaline phosphatase (ALP) level was measured using a BCIP/NBT ALP Color Development Kit (Beyotime, China) and Alizarin Red S (ARS) staining was done using ARS solution (Beyotime, China). The stained samples were examined under a stereomicroscope (ECHO Revolve, United States), and ALP activity was measured using an ALP assay kit (Beyotime, China). The calcification on each scaffold was assessed semi-quantitatively by dissolving the calcium nodules with 10% cetylpyridinium chloride and measuring the absorbance at 540 nm.

## 3 Results and discussion

### 3.1 Fabrication and characterization of hierarchical zeolite coatings

**FIGURE 1:**
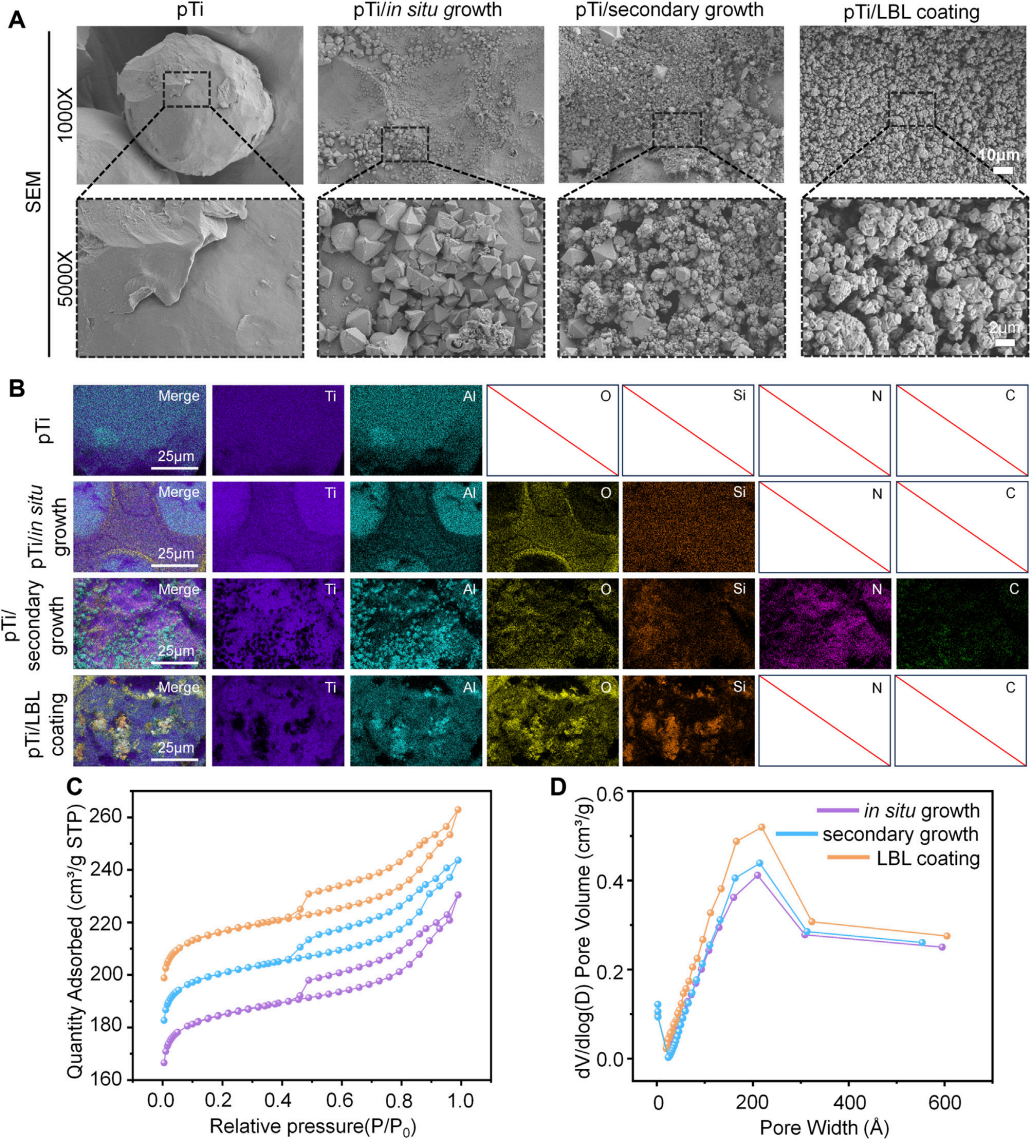
Characterization of hierarchical zeolite coatings. **(A)** SEM images of the surface morphology of pTi, pTi-*in situ* growth, pTi-secondary growth, and pTi-LBL coating samples. **(B)** The energy spectrum analysis of the different samples. **(C, D)** N_2_ adsorption-desorption isotherms and pore size distribution of the pTi, pTi-*in situ* growth, pTi-secondary growth, and pTi-LBL coating samples.

In this study, we employed *in situ* growth and secondary growth methods to create zeolite coatings on the surface of pTi. Subsequently, a dealumination reaction was performed to construct hierarchical zeolite coatings. Additionally, we successfully applied the LBL electrostatic self-assembly technique to coat hierarchical zeolite coatings onto pTi surfaces. As shown in Figure 1A, the untreated pTi surface, as observed through SEM, appeared smooth, while the coated pTi surfaces of the various coating groups exhibited different degrees of zeolite coatings. In particular, the distribution of hierarchical zeolites in the pTi-*in situ* growth group appeared less dense than the other coating groups, whereas the uniformity of multi-level, porous, molecular sieve hierarchical zeolite distribution in the pTi-secondary growth group was significantly improved. This improvement can be attributed to the secondary growth, which involves the prior uniform seeding of a layer of zeolite crystals on the substrate to serve as nuclei for crystal growth under hydrothermal conditions (Kuzniatsova et al., 2008). Subsequently, the substrate with the seeded layer is immersed in the growth mother liquor for cross-linking growth followed by modification with NH_4_HF_2_, resulting in a denser hierarchical zeolite coatings compared to the *in situ* growth group (Algieri and Drioli, 2021; Xiaofei et al., 2022). Simultaneously, we observed that the pTi surface treated using the LBL coating method exhibited a uniform morphology, with multi-level, porous molecular sieves evenly covering the substrate material’s surface, demonstrating the best performance among the three coating treatments. In LBL assembly methods, the process of charge neutralization and subsequent resaturation, triggered by the adsorption of counterionic component materials onto a charged surface, leads to charge inversion. This phenomenon facilitates the alternating adsorption of cationic and anionic samples as coatings (Ariga et al., 2019). Hence, we can achieve the construction of more uniformly continuous hierarchical zeolite coatings by varying the number of cycles of adsorption processes for cationic (PDDA) and anionic (hierarchical zeolite) samples.

To further analyze the effect of different hierarchical zeolite coating methods on the elemental composition of pTi, we performed surface elemental composition analysis on untreated pTi and coated pTi samples. As depicted in the EDS elemental analysis (Figure 1B), all four groups exhibited the presence of Ti and Al, which are intrinsic components of the substrate material. Notably, the coating groups contained elements O and Si, consistent with the primary constituents of hierarchical zeolites. The pTi-secondary growth group also featured N and C elements, attributable to the PDDA cationic solution used during the preseeding of the surface with crystal nuclei. Although the pTi-LBL coating group also employed a PDDA solution, the high-temperature calcination process performed as the final step effectively removed the organic components PDDA and PAA from the coating. Furthermore, the N2 adsorption-desorption isotherms revealed that when the relative pressure P/P_0_ was less than 0.4, a significant increase in adsorption indicated the presence of numerous microporous structures within the materials. As the relative pressure P/P_0_ continued to increase within the range of 0.4–1.0, all three groups of hierarchical zeolite coatings exhibited H4 type hysteresis loops, signifying the successful introduction of mesoporous structures within the materials (Figure 1C). Additionally, the pore-size distribution curves obtained from the Barret-Joyner-Halenda analysis further confirmed the successful construction of hierarchical zeolite coatings (Figure 1D).

### 3.2 Stability performance of implants and coatings

**FIGURE 2:**
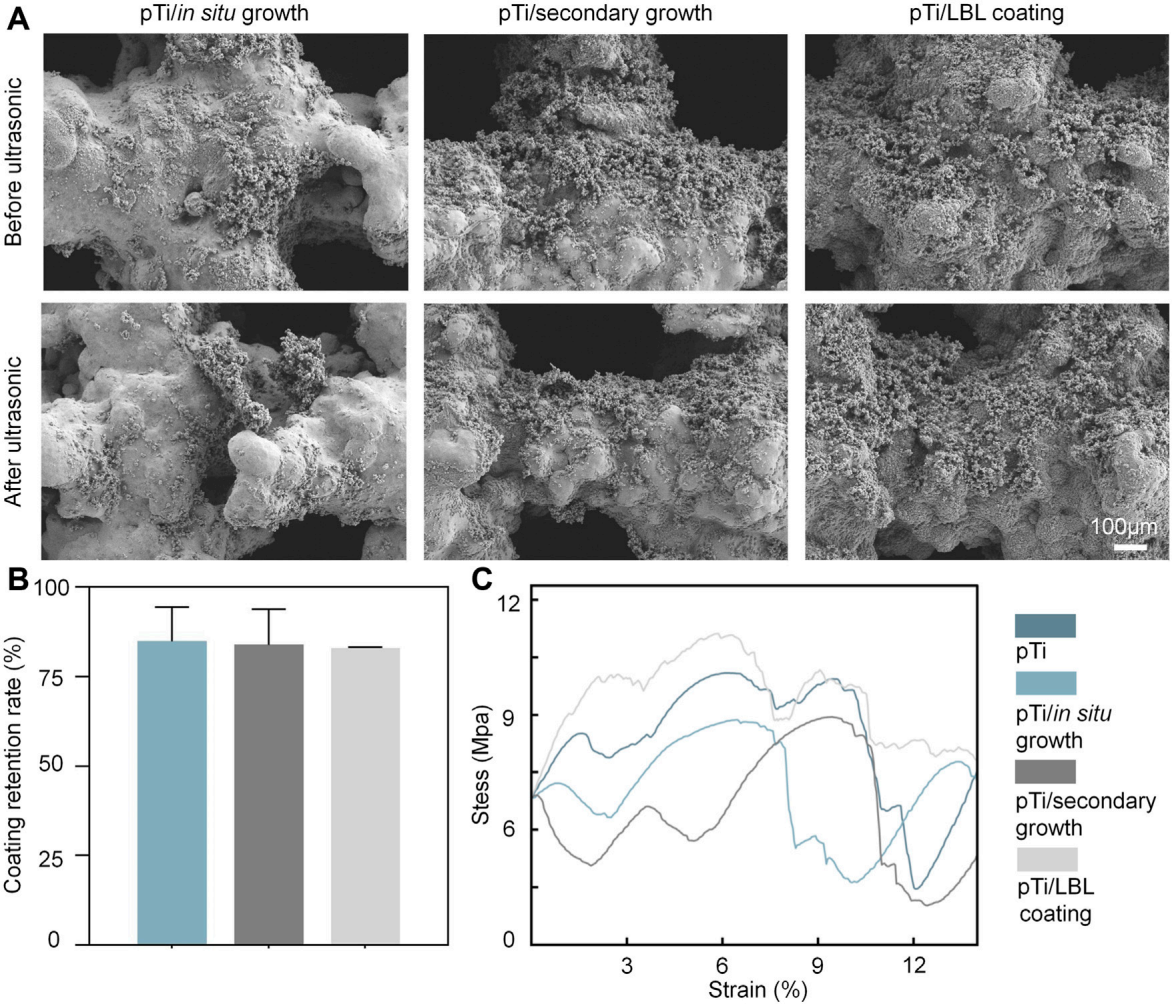
The stability performance of coatings and implants. **(A)** SEM images of the surface morphology of the pTi-*in situ* growth, pTi-secondary growth, and pTi-LBL coating samples before and after ultrasonic treatment. **(B)** The coating retention rate of the pTi-*in situ* growth, pTi-secondary growth, and pTi-LBL coating samples. **(C)** The compressive stress–strain curve of each group (*n* = 3).

This study primarily focused on the investigation of stability, which can be categorized into two main aspects: the adhesion stability of the implant interface coating and the mechanical stability of the entire implant. Our assessment of stability involved subjecting each coating sample to ultrasonic treatment at 50 kHz and subsequent SEM observations to evaluate the extent of coating retention before and after ultrasonication. As shown in Figures 2A, B, the coating retention rate for all three methods remained above 75% after ultrasonic treatment, with no significant differences among the three groups. Sufficient adhesion of the coating is essential for the successful functionality of coated implants in physiological environments, as previously highlighted by Sharifi et al. (Sharifi et al., 2018).

Furthermore, implants must meet certain criteria in terms of mechanical strength, biocompatibility, and structural stability to achieve optimal biological functionality (Mehwish et al., 2022). Among these criteria, mechanical stability is regarded as the fundamental prerequisite for implants to perform their intended biological functions (Huang et al., 2017). We conducted compression mechanical tests on pTi, pTi-*in situ* growth, pTi-secondary growth, and pTi-LBL coating samples using a universal testing machine. As depicted in Figure 2C, the pTi-LBL coating sample exhibited superior compressive deformation resistance compared to the other groups. This enhancement in mechanical performance can be attributed to the 500°C annealing process applied during the fabrication of this group of samples. In addition to eliminating organic compounds, this annealing process promotes dynamic recrystallization of the titanium alloy, a factor highlighted by HIDA et al. (Hida et al., 2013), who pointed out that heat treatment at 500°C optimizes the Young’s modulus and tensile strength of titanium alloys. Thus, our investigation of implant and coating stability in this segment confirms that the LBL-coating method exhibits superior overall performance.

### 3.3 *In vitro* biocompatibility of coatings

**FIGURE 3:**
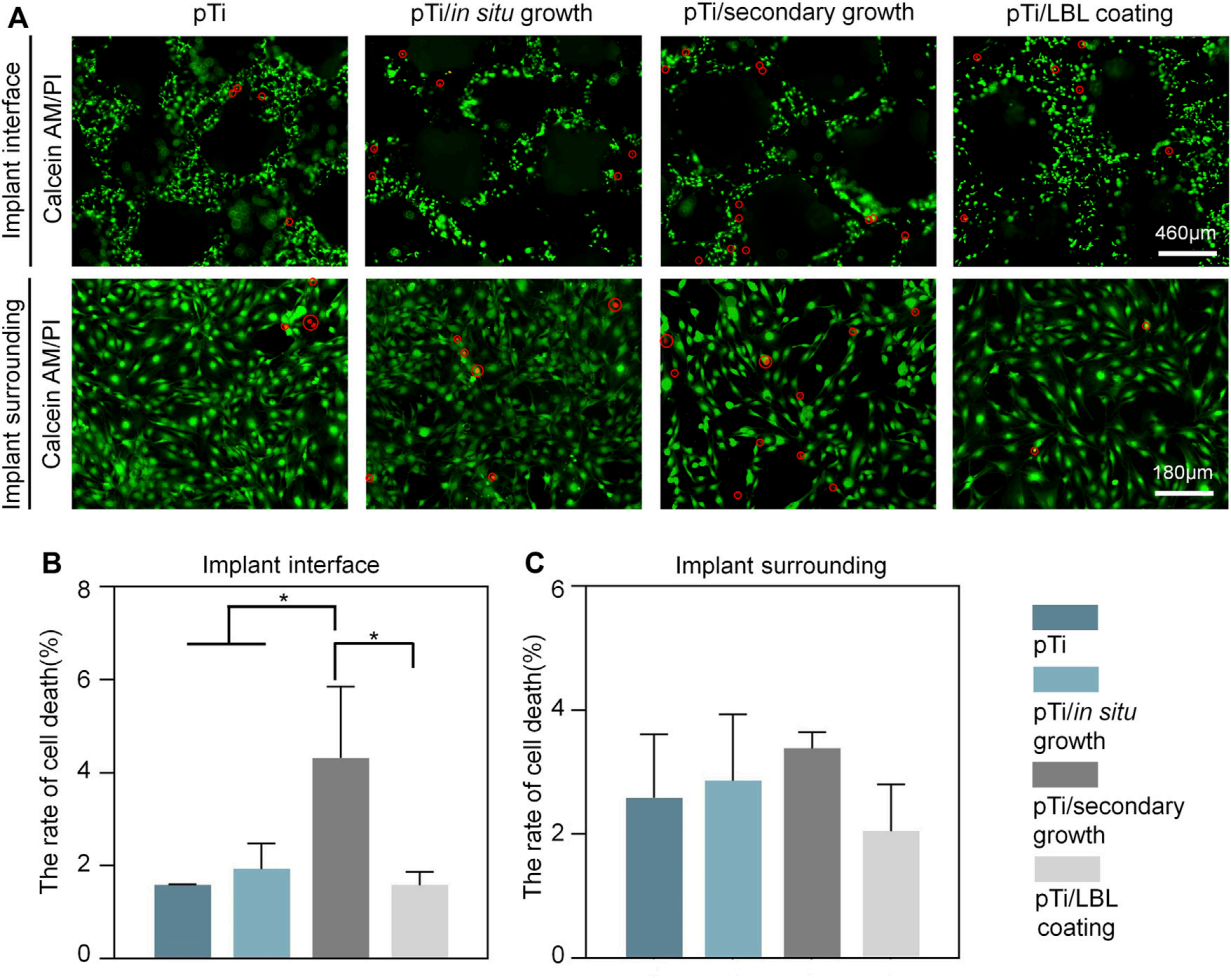
Assessment of *in vitro* biocompatibility in different coatings. **(A)** Calcein-AM/PI staining of MC3T3 cells after being cultured on pTi, pTi-*in situ* growth, pTi-secondary growth, and pTi-LBL coating samples. **(B,C)** Cell death rates of different coatings in the conditions of the implant interface and the surrounding environment (*n* = 3, *indicates significant differences between groups, *p < 0.05).

MC3T3 cells cultured in each group were stained with Calcein-AM/PI for the analysis of biocompatibility. As shown in Figure 3A, our analysis of live-dead staining on the surface of the implant coatings and of surrounding cells revealed that the number of living cells with green fluorescence was obviously more than the dead cells with red fluorescence in each group. Cell death rate, which represents the proportion of dead cells, was assessed using ImageJ. The cell death rates of the pTi, pTi-*in situ* growth, pTi-secondary growth, and pTi-LBL coating samples were lower than 10%, both in the implant interfaces and the environment surrounding the implants (Figures 3A, B).

Nonetheless, we noted a significantly elevated cell mortality rate on the surface of the pTi-secondary growth implants in comparison to the other sample groups. This phenomenon can be ascribed to the use of the PDDA solution during crystal seeding on the implant surface, leading to a higher PDDA content on the implant surface, thereby exerting an adverse influence on the cell viability of MC3T3 cells. Tang et al. (Tang et al., 2013) have also reported the dose-dependent toxicity of PDDA and its inclusion as a coating component may potentially impact biocompatibility. In contrast, although the LBL-coating samples similarly employed polymer solutions containing PAA and PDDA, the subsequent high-temperature calcination effectively eliminated any incorporated organic substances, thereby preserving biocompatibility without any adverse effects.

### 3.4 Effect of coating microstructure on cellular behavior

**FIGURE 4:**
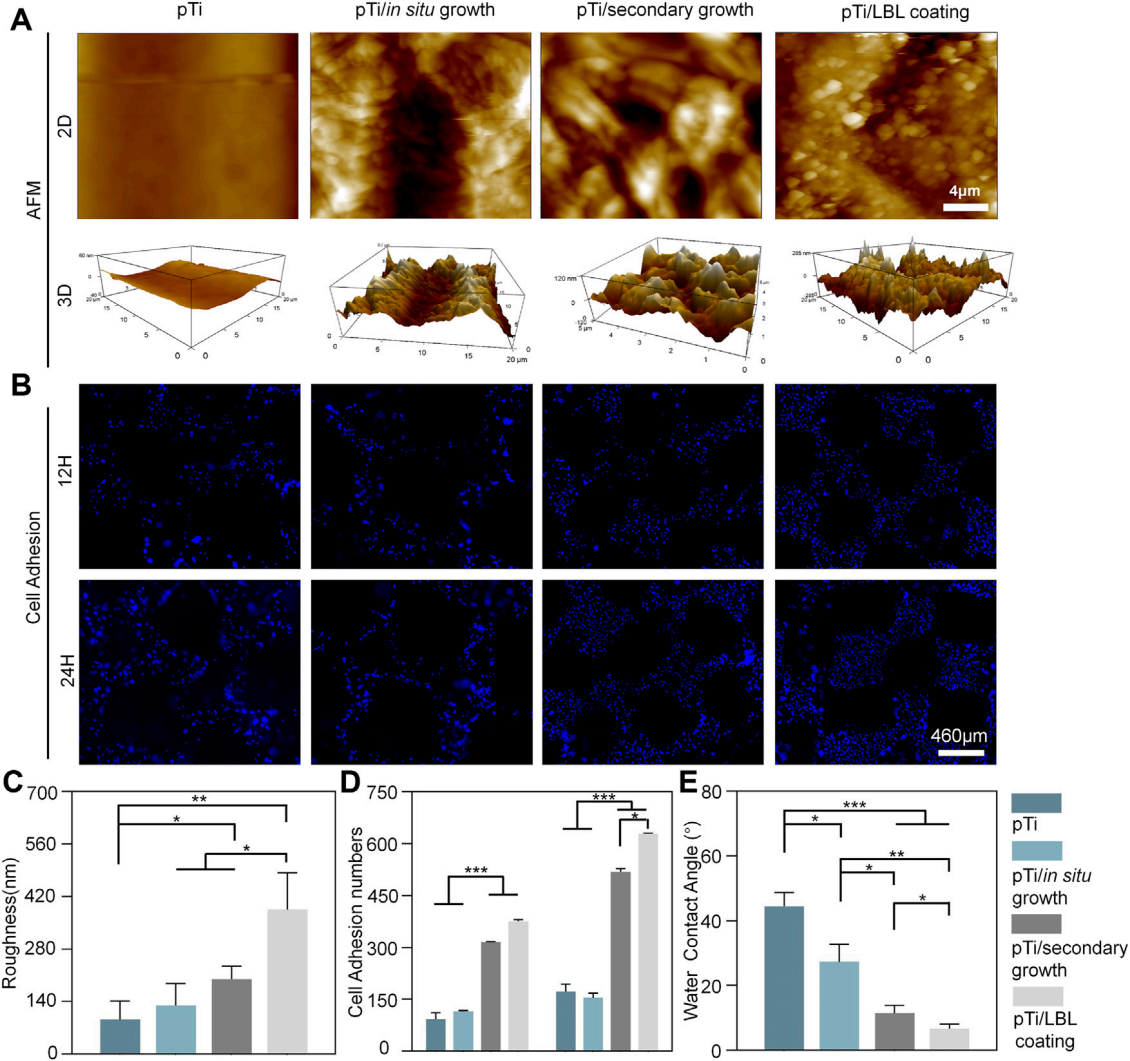
Surface roughness, hydrophilicity, and cell adhesion of the implants. **(A)** AFM micrographs of pTi, pTi-*in situ* growth, pTi-secondary growth, and pTi-LBL coating samples. **(B)** DAPI staining of MC3T3 cells after being cultured on different samples. **(C)** Surface roughness and cell adhesion **(D)** analysis of different samples. **(E)** Water contact angle measurement of the hydrophilicity of the four samples. (*n* = 3, *, ** and*** indicate *p* < 0.05, <0.01, and <0.001, respectively).

AFM analysis revealed that untreated pTi and pTi-*in situ* growth exhibited relatively low surface roughness, whereas pTi-secondary growth, and pTi-LBL coating had higher surface roughness (Figure 4A). The surface roughness of pTi-LBL coating, in particular, showed a significant increase. Statistical analysis of the pTi-LBL coating sample surfaces further confirmed this observation (Figure 4C). LBL assembly technology is a method that involves assembling molecular layers by utilizing intermolecular forces such as electrostatic attraction, hydrogen bonds, and covalent bonds. This process yields well-structured, stable, and functionally specialized molecular assemblies between these layers (McFerran et al., 2022). Furthermore, LBL technology provides versatility in terms of substrate materials, demonstrates remarkable adaptability, and is not limited by the substrate material’s type, size, or shape. Combining the results from Figures 1A, 4, it can be inferred that the LBL-coating method enables a more uniform and dense deposition of hierarchical zeolites on the pTi surface, which is important for the subsequent bioactive functionality of the coatings.

Cell adhesion of MC3T3 cells on the implant surfaces was assessed through DAPI staining. As shown in Figures 4B, D, there was an overall increasing trend in cell adhesion from 12 to 24 h. At both 12 and 24 h time points, the number of adherent MC3T3 cells on pTi-secondary growth and pTi-LBL coatings was significantly higher than the other two groups. However, at the 12 h time point, there was no significant difference between the pTi-secondary growth and pTi-LBL coating groups. In contrast, at the 24 h time point, a significant difference in the number of adherent cells was observed between the pTi-LBL coating and the pTi-secondary growth groups. This result may be attributed to the denser and more uniform spatial gradient structure on the surface of pTi-LBL coating implants, which is more favorable for promoting cell adhesion. This observation is consistent with the findings of (Qiao et al., 2020), who pointed out that surface microtopography can influence cell adhesion, cell extension, and cell cytoskeleton maintenance.

A water contact angle experiment was conducted to measure the hydrophilicity and confirm the coating effect (Figure 4E). The contact angle between the pTi sample and deionized water is approximately 44.47° ± 4.25°, significantly exceeding that of the other groups. Notably, the hydrophilicity of the pTi-LBL coating (6.67° ± 1.40°) group exhibits remarkable superiority compared to the pTi-*in situ* growth (27.3° ± 5.45°) and pTi-secondary growth (11.4° ± 2.5°) groups. This pronounced hydrophilicity can be chiefly attributed to the tetrahedral silicon-oxygen structure of the molecular sieve and the presence of abundant micro- and mesoporous structures, collectively resulting in a highly hydrophilic coating (Liu et al., 2018). An additional contributing factor is the surface microstructure alteration, particularly the augmented surface roughness, which further enhances the implant’s surface wettability (Beltrán et al., 2022). Therefore, the contact angle between the sample and deionized water significantly decreased after coating, promoting favorable conditions for the initial cell attachment (Jiawen et al., 2021).

**FIGURE 5:**
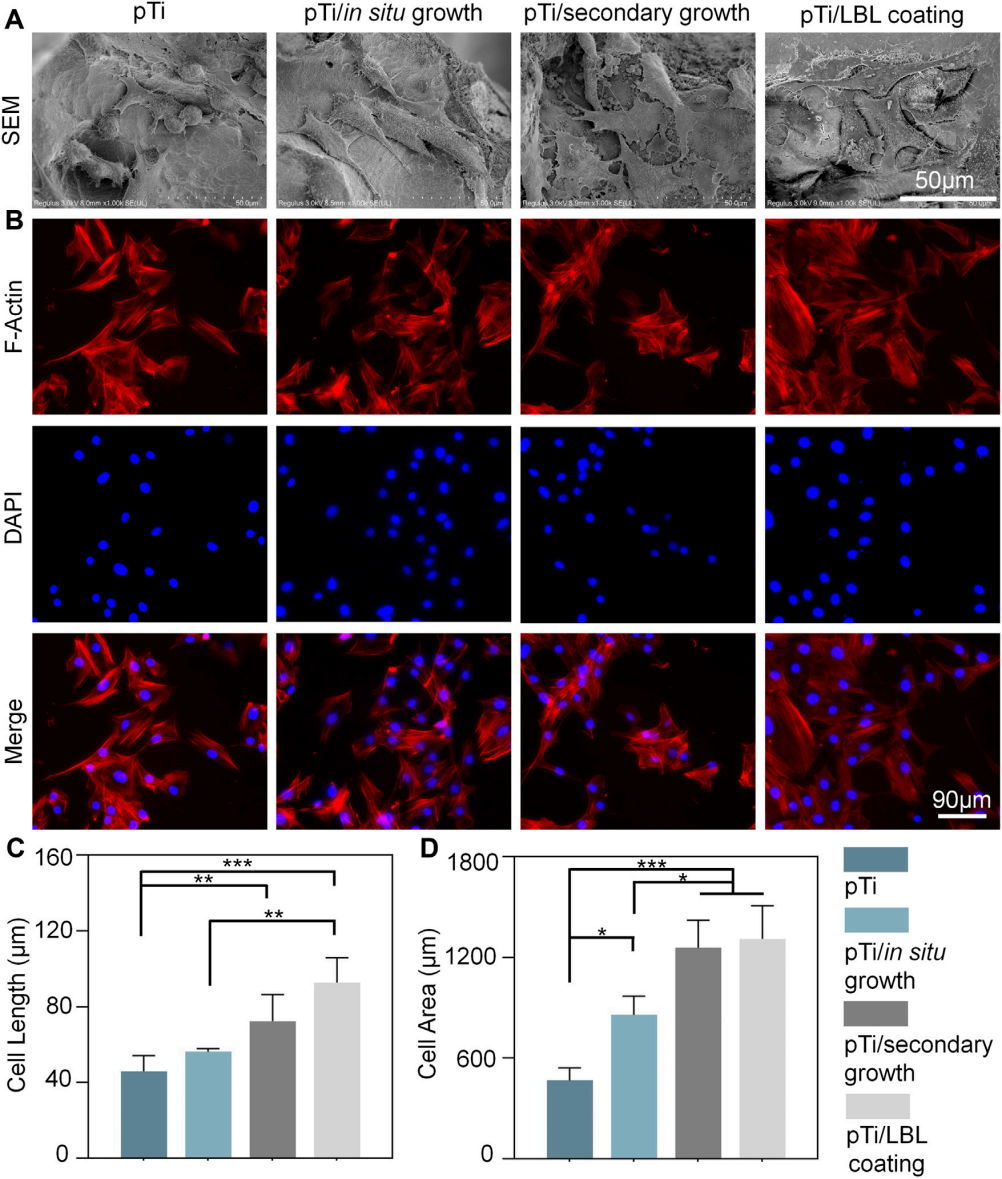
The impact of coating microstructure on the morphology of MC3T3 cells. **(A)** SEM images of adherent MC3T3-E1 cells on pTi, pTi-*in situ* growth, pTi-secondary growth, and pTi-LBL coating samples. **(B)** F-actin staining of MC3T3 cells after being cultured on different samples. **(C, D)** The related cellular length and area of SEM images were measured by using ImageJ software (*n* = 3, * indicating significant differences between groups, *p < 0.05, **p < 0.01, ***p < 0.001).

Following this, MC3T3 cells were seeded onto the implant surfaces and SEM analysis performed after 3 days. At this juncture, the cells were in a state of active proliferation and migration, enabling us to evaluate their adhesive morphology on the samples. Figure 5A showcases SEM images of cells adhered to different scaffold materials. It is apparent from these electron micrographs that cells adhering to the various scaffold materials displayed a spindle-shaped morphology, consistent with the typical appearance of MC3T3 cells. However, cells on the pTi and pTi-*in situ* growth scaffold surfaces appeared relatively isolated, while those on the surfaces of pTi-secondary growth and pTi-LBL coating groups displayed more interconnected cells. Additionally, distinct cellular pseudopodia extending from the cells were clearly observable, indicating that the cells were in an active state of proliferation and migration. These results were confirmed by F-actin staining, which marked the cell morphology of MC3T3 cells (Figure 5B). A statistical analysis of SEM cell images using ImageJ revealed that both the cell diameter and area of adherent cells on the pTi-secondary growth and pTi-LBL coating groups were significantly greater than those of the adherent cells of the other two groups (Figures 5C, D).

### 3.5 Osteogenic differentiation of MC3T3 cells *in vitro*

**FIGURE 6:**
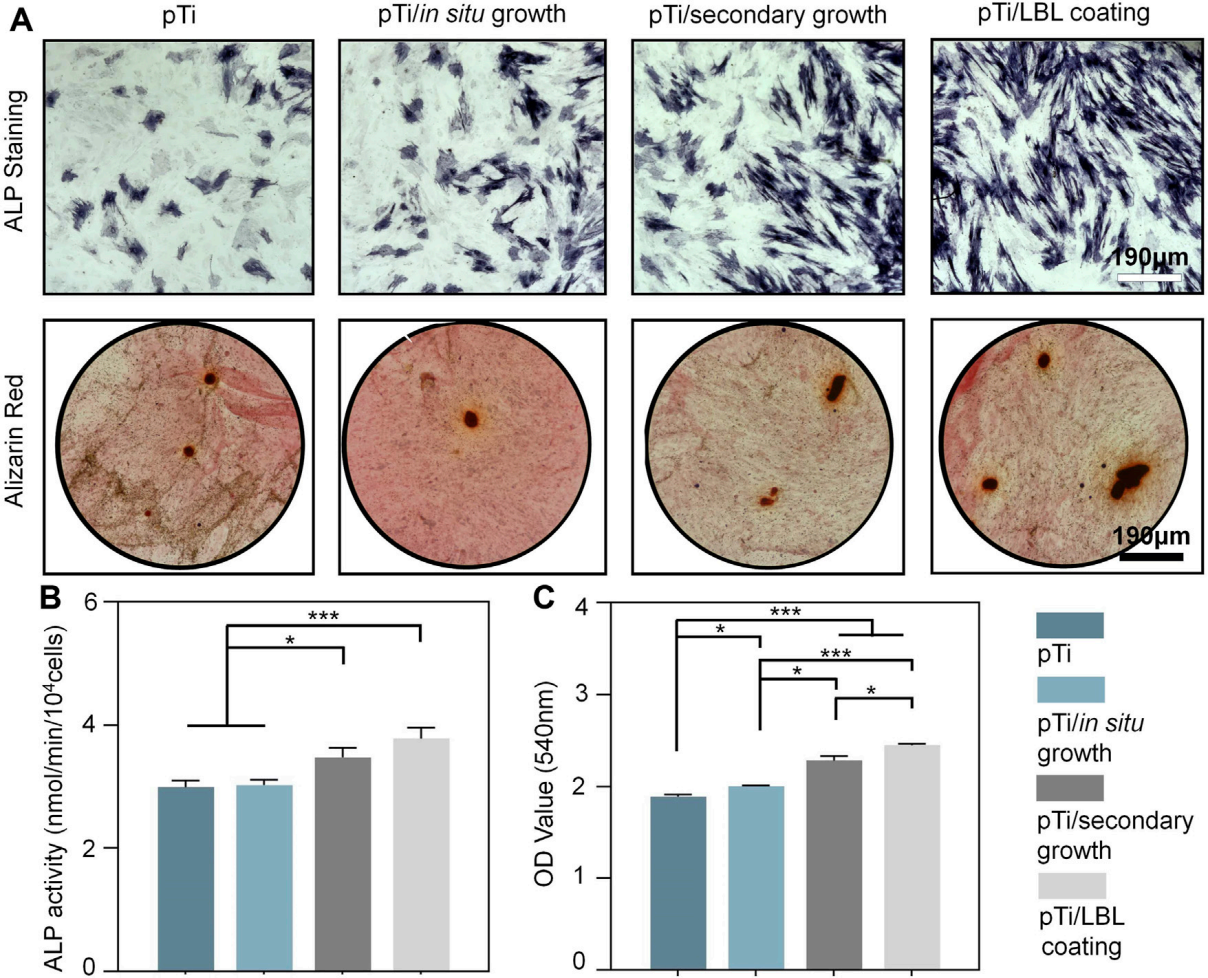
Effect of coatings on osteogenic differentiation. **(A)** ALP staining was conducted after a 7-day incubation of MC3T3 cells with pTi, pTi-*in situ* growth, pTi-secondary growth, and pTi-LBL coating samples. Alizarin Red staining was performed after co-culturing the cells with each group for 14 days. **(B)** Enzyme activity analysis of ALP was carried out following a 7-day co-culture of MC3T3 cells with each group. **(C)** Semi-quantitative analysis was based on Alizarin Red staining (*n* = 3, * indicating significant differences between groups, *p < 0.05, **p < 0.01, ***p < 0.001).

The osteogenic differentiation potential of pTi, pTi-*in situ* growth, pTi-secondary growth, and pTi-LBL coating samples was assessed through ALP enzyme activity, ALP staining, and Alizarin Red staining. ALP is an early biological marker for extracellular matrix (ECM) maturation in the osteogenic differentiation process, which is secreted in a vesicular form and is involved in ECM mineralization (Mao et al., 2021). As shown in Figure 6A, after 7 days of culture, the purple-stained areas in the pTi-LBL coating and pTi-secondary growth groups were significantly higher than in the other two groups, indicating higher ALP content. Subsequently, this result was further confirmed by the ALP enzyme activity assay, which showed that the ALP enzyme activity in the pTi-LBL coating group was higher than in the pTi-secondary growth group (Figure 6B).

The deposition of minerals is a hallmark of mature osteoblasts and serves as an indicator of the mineralization phase of the ECM during MC3T3-E1 cell differentiation (Cui et al., 2014). In this study, the mineralization capacity of each group was characterized through Alizarin Red staining. After 14 days of osteogenic induction, significant calcium nodule deposition was observed in the pTi-LBL coating and pTi-secondary growth groups. Further semi-quantitative analysis confirmed that the absorbance of stained calcium nodules in the pTi-LBL coating group (2.45 ± 0.02) was significantly higher than that in the pTi (1.89 ± 0.02), pTi-*in situ* growth (2.00 ± 0.01), and pTi-secondary growth (2.28 ± 0.05) groups (*p* < 0.05) (Figure 6C). These findings indicate that the interface constructed using the LBL coating method can enhance osteogenic capabilities.

The biomimetic environment for cells can enhance cell adhesion, proliferation, and later development, ultimately promoting osteogenic cell behavior and contributing to bone tissue regeneration. This regulatory capacity can be influenced by the surface characteristics of implants, including surface morphology, roughness, and hydrophilicity (Szewczyk et al., 2019). The surface microstructure provides topographical cues that regulate cell differentiation or maintain their multipotency, and it can either enhance or diminish cell adhesion (Zhou et al., 2021). Srivas et al. (Srivas et al., 2019) pointed out that the rough surface resulting from hierarchical surface morphology significantly influences cell cytoskeletal structure by altering cell signaling pathways, thereby affecting cell spreading and proliferation on Ti6Al4V. Furthermore, roughness results in increased surface energy and hydrophilicity, accelerating enhanced initial protein adsorption and promoting cell interactions at the implant interface (Petrini et al., 2021). Hence, cell adhesion can be controlled by surface properties, and the combination of surface charge distribution and material chemistry can further modulate cell adhesion, migration, proliferation, and differentiation (Kulkarni et al., 2015). Surface morphology can also influence the osteogenic differentiation capability of cells (Gabler et al., 2015). However, most of the research on surface-layering structures has primarily focused on the “microporous and macroporous” levels. In contrast, the “microporous, mesoporous, and macroporous” layered spatial gradient structure investigated in this study holds more potential as a surface modification strategy for regulating osteogenic capability and bone integration. In this study, we have confirmed that coating the implant surface using the LBL assembly method promotes cell adhesion and spreading while significantly enhancing the osteogenic differentiation capability of MC3T3 cells.

## 4 Conclusion

In summary, this is the first report of the successful development of a hierarchical zeolite coating with a “microporous-mesoporous-macroporous” spatial gradient structure on the surface of pTi. Our research involved a thorough comparative analysis of various coating fabrication methods, incorporating chemical, physical, and biological assessments. Notably, the LBL assembly method yielded coatings distinguished by their uniformity, density, excellent adhesion, and compression resistance, all of which are pivotal for ensuing biological functionality. This innovative LBL-fabricated coating exhibited remarkable biocompatibility with MC3T3-E1 cells. Furthermore, LBL-coated samples showcased heightened surface roughness and hydrophilicity, augmenting the adhesion, proliferation, and spreading capabilities of MC3T3-E1 cells. Lastly, the pTi-LBL coating samples, characterized by their uniformly distributed spatial gradient coating, considerably promoted osteogenic differentiation. In conclusion, the hierarchical zeolite coating, established via the LBL assembly method, holds considerable clinical significance and establishes a crucial foundation for future initiatives in crafting bioactive interfaces.

## Data Availability

The original contributions presented in the study are included in the article/supplementary material, further inquiries can be directed to the corresponding authors.
